# Mitigating methane emissions and global warming potential while increasing rice yield using biochar derived from leftover rice straw in a tropical paddy soil

**DOI:** 10.1038/s41598-024-59352-5

**Published:** 2024-04-15

**Authors:** Saowalak Somboon, Benjamas Rossopa, Sujitra Yodda, Tanabhat-Sakorn Sukitprapanon, Amnat Chidthaisong, Phrueksa Lawongsa

**Affiliations:** 1 Department of Soil Science and Environment, Faculty of Agriculture, Khon Kaen University, Khon Kaen, 40002 Thailand; 2 Soil Organic Matter Management Research Group, Khon Kaen University, Khon Kaen, 40002 Thailand; 3 Prachin Buri Rice Research Center, Rice Department, Ministry of Agriculture and Cooperatives, Ban Sang, Prachin Buri 25150 Thailand; 4 Program on System Approaches in Agriculture for Sustainable Development, Department of Agricultural Extension and Agricultural Systems, Faculty of Agriculture, Khon Kaen University, Khon Kaen, 40002 Thailand; 5 The Joint Graduate School of Energy and Environment, King Mongkut’s University of Technology Thonburi, Bangkok, 10140 Thailand

**Keywords:** Climate sciences, Environmental sciences

## Abstract

The sustainable management of leftover rice straw through biochar production to mitigate CH_4_ emissions and enhance rice yield remains uncertain and undefined. Therefore, we evaluated the effects of using biochar derived from rice straw left on fields after harvest on greenhouse gas emissions, global warming potential (GWP), and rice yield in the paddy field. The experiment included three treatments: chemical fertilizer (CF), rice straw (RS, 10 t ha^−1^) + CF, and rice straw-derived biochar (BC, 3 t ha^−1^ based on the amount of product remaining after pyrolysis) + CF. Compared with CF, BC + CF significantly reduced cumulative CH_4_ and CO_2_ emissions, net GWP, and greenhouse gas emission intensity by 42.9%, 37.4%, 39.5%, and 67.8%, respectively. In contrast, RS + CF significantly increased cumulative CH_4_ emissions and net GWP by 119.3% and 13.8%, respectively. The reduced CH_4_ emissions were mainly caused by the addition of BC + CF, which did not increase the levels of dissolved organic carbon and microbial biomass carbon, consequently resulting in reduced archaeal abundance, unlike those observed in RS + CF. The BC + CF also enhanced soil total organic carbon content and rice grain yield. This study indicated that using biochar derived from leftover rice straw mitigates greenhouse gas emissions and improves rice productivity in tropical paddy soil.

## Introduction

Rice (*Oryza sativa* L.) is the most popular and widely cultivated crop in Thailand and occupies more than 60% of the farmed land in the northeastern region^1^. The paddy soils in the northeast are generally less fertile due to their high sand content, low soil organic carbon (SOC) content, acidity, and very low to moderately low cation exchange capacity^2^. Importantly, rice cultivation has been poorly managed over the long term in the northeast, and residual rice straw is usually burned in open fields or removed after each harvest^3^. This practice leads to a decrease in SOC content, resulting in the degradation of paddy soils^4,5^. A sustainable solution to this problem entails returning crop straw to fields, which can improve soil carbon (C) sequestration, soil fertility, and rice yields^6^. However, rice straw easily decomposes and releases C substrates such as dissolved organic carbon (DOC), labile organic carbon (LOC), microbial biomass carbon (MBC), acetate, and H_2_/CO_2_, providing a conducive environment for methanogenic archaea that contribute to methane (CH_4_) production^7,8^. Biogenic CH_4_ production involves a diverse microbial community of bacteria and archaea^9^. CH_4_ is generated by methanogenic archaea in anaerobic environments; nevertheless, methanotrophic bacteria oxidize some of this gas before its release into the atmosphere^10^. This suggested that labile C fractions, archaeal abundances, and bacterial abundances in soils were the primary factors regulating CH_4_ emissions under anaerobic conditions.

Biochar is a C-rich solid, with high porosity, a large surface area, a high capacity for cation exchange, and low-cost pyrolysis produced from renewable waste products e.g., lignocellulose chips, rice straw, and dried leaves^11,12^. Previous studies have demonstrated that biochar can be applied to croplands as an amendment to improve C sequestration, increase crop productivity, and reduce emissions of greenhouse gases (GHGs) such as CH_4_ and carbon dioxide (CO_2_)^13–15^, especially CH_4_ emissions from paddy soils^16–19^. The addition of biochar to paddy soils has been found to reduce CH_4_ emissions by altering microbial community structure and activity, reducing the availability of C sources for methanogenesis, and enhancing C sequestration in soil^20,21^. However, biochar can also increase soil CH_4_ emissions^22–24^, which depends on biochar properties, application rate, and soil conditions. This emphasized the necessity for further studies of CH_4_ emissions from soil with biochar amendments.

Recently, several studies have focused on applying biochar to paddy soils at high rates, ranging from 10 to 80 t ha^−1^^25–29^. A higher rate of biochar application resulted in a reduction in cumulative CH_4_ emissions^27^. Conversely, Yang et al*.*^29^ suggested that applying biochar at a lower rate is optimal for reducing GHG emissions in subtropical paddy fields. Nevertheless, some recent studies have shown that biochar amendment of paddy soil at either a low (2.8 t ha^−1^) or high (22.5 t ha^−1^) rate can mitigate CH_4_ emissions^17,18^. Moreover, high biochar application rates (≥ 17 t ha^−1^) lead to crop yield reductions by reducing nutrient supplies^29–31^. In contrast, Chen et al*.*^28^ showed that rice yield increased with increasing biochar application (0, 20, and 40 t ha^−1^). Applying biochar at a high rate may not be environmentally sustainable or cost-effective due to the large amount of raw materials needed. Hence, using biochar produced from rice straw at a rate corresponding to the annual output could serve as a practical approach to enhancing both sustainable straw management and economic advantages. Previous studies have suggested that applying biochar produced from the pyrolysis of leftover rice straw reduces CH_4_ emissions in subtropical rice paddy fields^17,18^. However, rice straw yields depend on rice cultivation practices and varieties; thus, the yield of biochar from leftover rice straw varies. Furthermore, how biochar addition affects GHG emissions and rice yields in paddy fields has been unclear. Hence, we have conceptual idea to convert rice straw residues, estimated at approximately 10 t ha^−1^ according to the remaining straw in the fields^32,33^ into biochar, and subsequently apply the biochar at a rate based on the pyrolysis product yield into the soil. We selected this specific application rate with the goal of sustainably utilizing rice straw to mitigate GHG emissions, enhance soil C sequestration, and improve rice productivity in tropical paddy fields.

Therefore, this study aimed to investigate the effects of biochar derived from leftover rice straw application on GHG emissions and rice yield and compare the results to those from leftover rice straw application in a tropical paddy field. Here, we applied 3 t ha^−1^ of rice straw-derived biochar, which was produced from 10 t ha^−1^ of leftover rice straw. Thus, the unique properties of biochar, like stable C structure, high porosity, and high cation exchange capacity, contribute to soil C sequestration and enhance fertility. We addressed the hypotheses that the addition of rice straw-derived biochar to paddy soil could reduce soil CH_4_ and CO_2_ emissions, net global warming potential (GWP), and greenhouse gas emission intensity (GHGI) by reducing archaeal abundance, influenced by a low labile C content in the soil, while simultaneously increasing rice grain yield. The objectives of this study were (1) to quantify the responses of soil CH_4_ and CO_2_ emissions to rice straw and rice straw-derived biochar applications in rice paddy fields, (2) to evaluate the effects of rice straw and rice straw-derived biochar applications on the net GWP, GHGI, and rice yield, and (3) to evaluate the effects of soil microbial abundance and biomass and soil chemical properties in regulating soil GHG emissions.

## Results

### Soil properties

**Figure 1:**
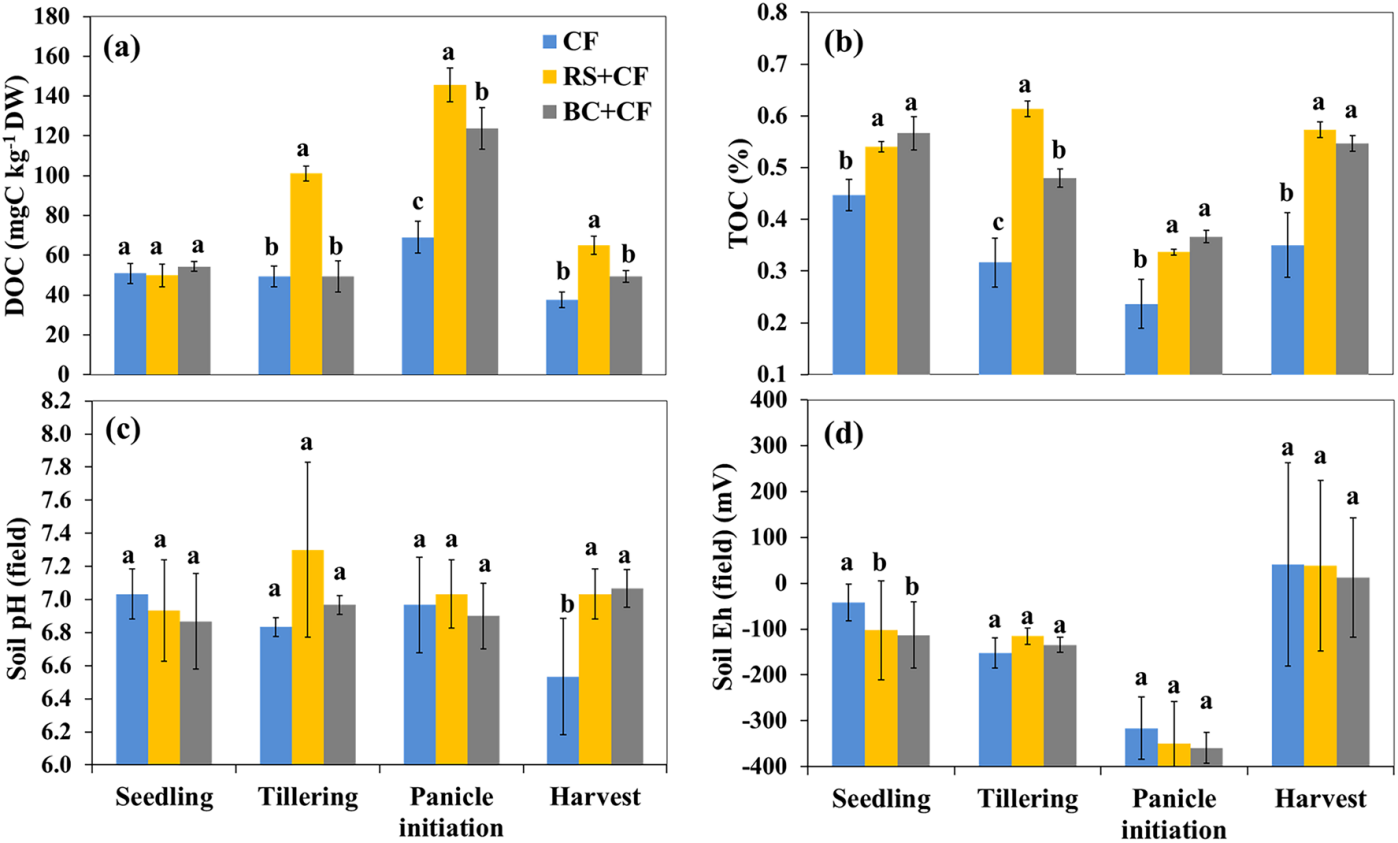
Means and standard deviation (error bar) of soil dissolved organic carbon (DOC) (**a**), total organic carbon (TOC) (**b**), pH (**c**), and redox potential (Eh) (**d**) levels under different treatments at different growth stages (*SL* seedling stage, *TL* tillering stage, *PI* panicle initiation stage, and *HV* harvest stage). Different letters indicate significant differences among treatments according to the LSD test with the confidence level set to 0.01.

Changes in the soil chemical properties during the rice growing season are shown in Fig. 1. The soil DOC content peaked in the tillering (TL) to panicle initiation (PI) stages in the RS + CF treatment, while it peaked in the PI stage in the other treatments. After the seedling (SL) stage, the RS + CF treatment resulted in the highest DOC content during the whole rice growing season (Fig. 1a). The soil TOC content in the RS + CF treatment increased at the TL stage and decreased at the PI stage. In contrast, the TOC content in the BC + CF and CF treatments gradually decreased after the SL stage to the PI stage. The highest TOC content was found in the RS + CF and BC + CF treatments during the whole rice growing season (Fig. 1b). The soil pH of the treatments ranged from 6.5 to 7.3 during the rice growing season and there were no significant differences among treatments (*p* < 0.01; Fig. 1c). The soil Eh exhibited similar trends among the cropping seasons and treatments (Fig. 1d).

### CH_4_ and CO_2_ emissions

**Figure 2:**
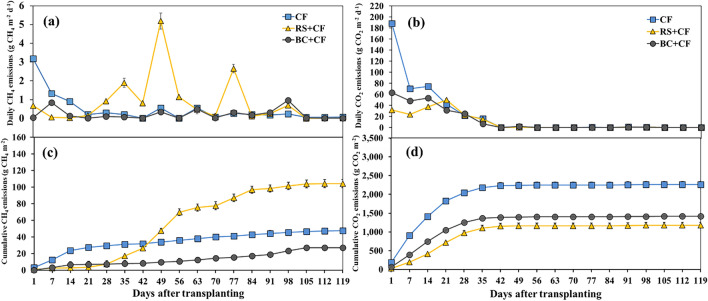
Means and standard deviation (error bar) of daily emissions of CH_4_ (**a**) and CO_2_ (**b**) and cumulative emissions of CH_4_ (**c**) and CO_2_ (**d**) in paddy soils treated with different treatments, including chemical fertilizer (CF), rice straw (RS) + CF, and rice straw-derived biochar (BC) + CF during the rice growing season.

Variation in daily CH_4_ emissions from all treatments throughout the rice growing season, as shown in Fig. 2a. The RS + CF treatment had higher CH_4_ emission during 28–56 days after transplanting (DAT) (during the TL stage) than the CF and BC + CF treatments. Consequently, differences between the cumulative CH_4_ emission trends of the soils treated with RS + CF and BC + CF during the rice growing season are shown in Fig. 2c. The cumulative CH_4_ emissions from the RS + CF treatment were significantly higher than those from the other treatments (*p* < 0.01; Table 1). In contrast, the BC + CF treatment had the lowest cumulative CH_4_ emissions. In the RS + CF treatment compared to those in the other treatments, the cumulative CH_4_ emissions increased sharply from the early rice season to 84 DAT (during the TL stage) and exhibited persistent and stable emission levels after 105 DAT (PI stage) to 119 DAT (harvest stage; HV). Unlike those in the BC + CF treatment, the cumulative CH_4_ emissions gradually increased from the SL to the HV stage and followed similar patterns in the CF treatment. The application of RS + CF significantly increased the seasonal cumulative CH_4_ emissions by 119.3%, whereas the BC + CF application significantly decreased the seasonal cumulative CH_4_ emissions by 42.9% compared with those of the CF treatment (*p* < 0.01; Table 1).

**Table 1:** Seasonal cumulative CH_4_ and CO_2_ emissions, global warming potential (GWP), and greenhouse gas intensity (GHGI) under different treatments in the paddy field.

Treatment	Cumulative CH_4_ emissions (kg ha^−1^ season^−1^)	% Reduction	Cumulative CO_2_ emissions (kg ha^−1^ season^−1^)	% Reduction	GWP of CH_4_ (kg CO_2_-eq ha^−1^)	GWP of CO_2_ (kg CO_2_-eq ha^−1^)	Net GWP (kg CO_2_-eq ha^−1^)	% Reduction	GHGI (kg CO_2_-eq t^−1^ GY^−1^)	% Reduction
CF	474.8b	–	22,660.4a	–	13,294.4b	22,660.4a	35,955.1b	–	5724.3a	–
RS + CF	1041.3a	119.3	11,773.4c	− 48.0	29,156.4a	11,773.4c	40,928.9a	13.8	3823.3b	− 33.2
BC + CF	270.9c	− 42.9	14,176.8b	-37.4	7,585.2c	14,176.8b	21,760.5c	− 39.5	1843.7c	− 67.8
F test	**		**		**	**	**		**	
C.V. (%)	4.9		2.4		4.9	2.4	3.5		5.3	

The different letters represent a significant difference (*p* < 0.01) among all the treatments.

In contrast to CH_4_ emissions, daily and cumulative CO_2_ emissions exhibited similar trends during the rice growing season and treatments (Fig. 2b,d). All treatments had a gradual decrease in CO_2_ emissions from 1 to 42 DAT (during the TL stage), and thereafter the values were relatively constant throughout the rice growing season (Fig. 2b). Therefore, soil cumulative CO_2_ emissions increased sharply after the transplantation of the rice crop to 42 DAT and exhibited persistent and stable emission levels until the HV stage (Fig. 2d). The cumulative CO_2_ emissions in the CF treatment were higher than those in the RS + CF and BC + CF treatments; moreover, the lowest cumulative CO_2_ emissions were observed in the RS + CF treatment. Compared with those of the CF treatment, the application of RS + CF and BC + CF significantly decreased the seasonal cumulative CO_2_ emissions by 48.0% and 37.4%, respectively (*p* < 0.01; Table 1).

### Soil microbial abundance and biomass

**Figure 3:**
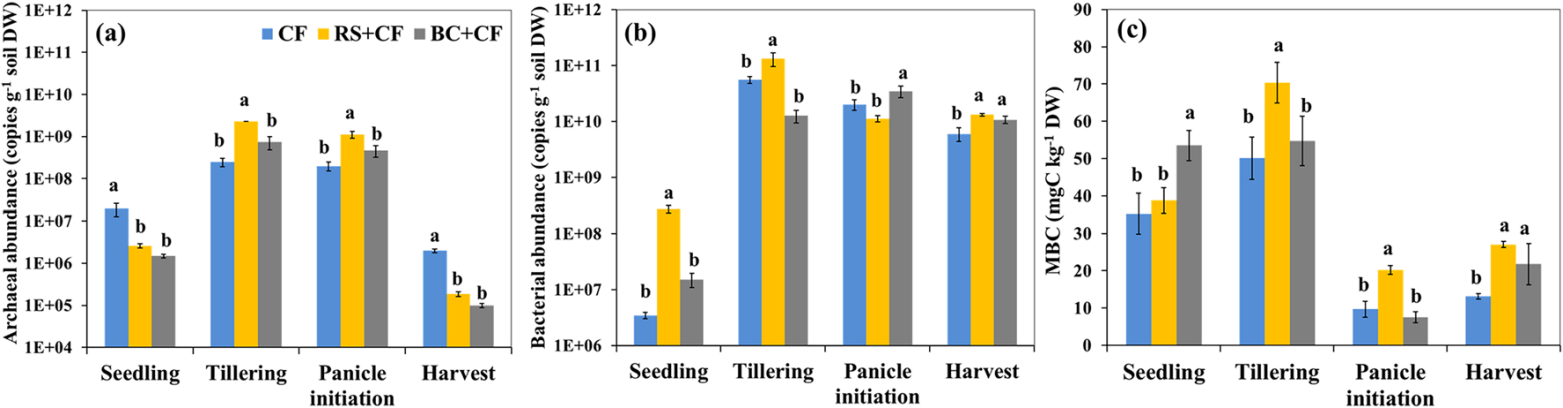
Means and standard deviation (error bar) of archaeal (**a**) and bacterial (**b**) abundances and microbial biomass carbon (MBC) (c) content in soil treated with different treatments at different growth stages of rice crop (seedling stage; SL, tillering stage; TL, panicle initiation stage; PI, and harvest stage; HV). Different letters indicate significant differences among treatments according to the LSD test, with the confidence level set to 0.01.

A similar pattern of archaeal abundance was observed for all treatments throughout the growing season (Fig. 3a). The highest archaeal abundance was observed during the TL and PI stages. The RS + CF treatment resulted in the highest archaeal abundance, while there were no significant differences among the BC + CF and CF treatments (*p* < 0.01). Similarly, the abundance of bacteria exhibited similar trends among the cropping seasons and treatments (Fig. 3b). The bacterial abundance sharply increased and reached its highest level at the TL stage and slightly decreased at the HV stage. At the TL stage, the highest bacterial abundance was found in the RS + CF treatment, and there were no significant differences among the BC + CF and CF treatments (*p* < 0.01). The soil MBC contents in the RS + CF and CF treatments increased at the TL stage, except for that in the BC + CF treatment. For all the treatments, the soil MBC content exhibited a sharp decrease at the PI stage. The highest MBC content was found in the RS + CF treatment during the TL and PI stages (Fig. 3c). During the TL stage, there were high levels of soil MBC and archaeal and bacterial abundances. The Pearson correlation analyses (Table 3) showed that CH_4_ emissions had a significantly positive relationship with soil archaeal abundance (*r* = 0.800, *p* < 0.01), bacterial abundance (*r* = 0.912, *p* < 0.001), and MBC (*r* = 0.931, *p* < 0.001) at the TL stage.

### Seasonal GWP and GHGI, and rice yield

The overall GWP and GHGI data for the paddy soil during the rice growing season are shown in Table 1. The RS + CF treatment had the highest net GWP, while the lowest net GWP was found in the BC + CF treatment. Compared to the CF treatment, the net GWP in the BC + CF treatment decreased by 39.5%, but that in the RS + CF treatment increased by 13.8%. As measured by rice yield, the GHGI was highest in the CF treatment and lowest in the BC + CF treatment. Additionally, the applications of RS + CF and BC + CF significantly increased the 1000-grain weight and grain yield compared to those of the plants treated with CF alone (*p* < 0.01; Table 2).

**Table 2:** Effects of rice straw and its derived biochar treatments on the growth and yield of the rice variety RD6.

Treatment	Number of tillers	Plant height (cm)	Straw yield (kg ha^−1^)	Filled grain percentage (%)	1000-Grian weight (g)	Grain yield (t ha^−1^)
CF	15.1a	229.9a	15,962.5a	90.7a	24.3b	6.3b
RS + CF	17.7a	221.5a	14,726.1a	92.3a	25.3a	10.6a
BC + CF	16.9a	215.1a	17,600.1a	93.5a	25.6a	11.8a
F test	ns	ns	ns	ns	**	**
C.V. (%)	11.6	4.1	8.0	4.0	0.9	6.4

The different letters represent a significant difference (*p* < 0.01) among all the treatments.

## Discussion

Converting rice straw to biochar, rather than directly incorporating it into paddy fields is a promising method for reducing CH_4_ emissions and increasing rice yield^25^. In the present study, compared to CF alone, the RS + CF amendment significantly increased seasonal cumulative CH_4_ emissions and net GWP, while significantly reduced seasonal cumulative CO_2_ emissions and GHGI (*p* < 0.01). In contrast, the BC + CF amendment significantly reduced seasonal cumulative CH_4_ emissions, cumulative CO_2_ emissions, net GWP, and GHGI (*p* < 0.01). Additionally, applications of RS + CF and BC + CF significantly increased grain yield compared to CF alone (*p* < 0.01).

The addition of BC + CF significantly reduced cumulative CH_4_ emissions, whereas the RS + CF amendment significantly increased cumulative CH_4_ emissions of the rice season (*p* < 0.01). We observed higher DOC and MBC contents and greater archaeal and bacterial abundances in the RS + CF treatment than in the BC + CF treatment, especially at the tillering stage. Moreover, CH_4_ emissions were significantly positively correlated with archaeal abundance (*r* = 0.800, *p* < 0.01), bacterial abundance (*r* = 0.912, *p* < 0.001), MBC content (*r* = 0.931, *p* < 0.001), and DOC content (*r* = 0.982, *p* < 0.001) in the tillering stage (Table 3). This was an indication that the increase in CH_4_ emissions in the RS + CF-treated soil was due to the high proportion of labile C components, such as cellulose (48.9%) and hemicellulose (29.2%) in the RS material (Table 4), which was able to stimulate C mineralization, leading to higher soil DOC and MBC contents. The increase in soil DOC and MBC contents stimulated archaeal and bacterial growth as C substrate sources, thereby promoting CH_4_ emissions, in agreement with the results obtained in a previous study^5,25,34^. Wang et al.^8^ reported that the concentrations of DOC and MBC were significantly greater (by 7.1–128.6%) in response to rice straw treatment than in the controls in a subtropical paddy field. The addition of labile C is likely to increase CH_4_ emission through the degradation of added C and may affect the degradation rate of native organic C^35^. In contrast to RS material, BC material contains high levels of recalcitrant C compounds, such as lignin (56.3%) and fixed C (40.7%) (Table 4), which are highly stable and beneficial for SOC accumulation^36,37^. The mineralization of soil organic matter (SOM) decreased when biochar was added, and the level of SOM increased^38^. Wang et al.^39^ also illustrated that biochar addition enhanced exogenous C sequestration by the soil, with a decomposition rate of biochar of only 5.7% year^−1^ in an intensive rice–wheat cropping system. In our study, compared with RS + CF, BC + CF addition suppressed C mineralization in soil, which was supported by enhanced soil TOC and reduced labile C fractions such as DOC and MBC. As a result, the BC + CF-treated soil had a lower archaeal abundance, resulting in lower CH_4_ emissions. Our findings were in agreement with previous studies showing that a reduction in soil DOC content as a result of biochar amendment is an important mechanism of methanogenic activity inhibition; thus, CH_4_ emissions were reduced^16,20^. Additionally, the availability of labile C substrates influences soil CH_4_ production^10^ by regulating microbial growth and activity. Huang et al*.*^27^ also reported that biochar addition decreased CH_4_ emissions, which may be due to decreased methanogenic archaeal abundance. Furthermore, Pan et al*.*^40^ suggested that soil microorganisms utilize more straw-C than biochar-C in paddy soil.

**Table 3:** Pearson’s correlation coefficients (*r*) for the different greenhouse gas emissions and soil properties in the different treatments at the tillering stage.

Pearson’s correlation	Archaea	Bacteria	DOC	MBC	TOC	pH	Eh
CH_4_	0.800**	0.912***	0.982***	0.931***	0.764*	0.515	0.533
CO_2_	− 0.650	− 0.697*	− 0.849**	− 0.779*	− 0.718*	− 0.401	− 0.538
DOC	0.804**	0.889**	1.000	0.895**	0.779*	0.398	0.499
MBC	0.718*	0.741*	0.896**	1.000	0.875**	0.441	0.609

**Table 4:** Chemical properties of the rice straw and rice straw-derived biochar used in the present study.

Property	Rice straw	Rice straw-derived biochar
pH (H_2_O)	6.8	8.9
%Total C	34.7	46.9
%Total N	0.6	0.5
Total P (mg kg^−1^)	361.5	1281.6
Total K (mg kg^−1^)	5,190.1	15,775.4
%Cellulose	48.9	30.9
%Hemicellulose	29.2	0.9
%Lignin	5.4	56.3
%Ash	19.5	25.1
%Volatile matter	65.3	32.2
%Fixed C	11.1	40.7

Compared to CF alone, the BC + CF treatment significantly reduced seasonal cumulative CH_4_ and CO_2_ emissions (*p* < 0.01; Table 1). Nevertheless, the soil DOC content and archaeal abundance were similar. Conversely, the TOC content in the BC + CF treatment was higher than that in the CF treatment. This indicated that the soil treated with BC + CF had low DOC availability, which suppressed microbial decomposition. Several previous studies suggested that BC can stabilize native SOC by forming organo-mineral complexes and through the sorption of DOC onto its surface and within pore spaces^41–44^. This leads to reduced organic decomposition, a substantial increase in C sequestration in soil^45^, and indirect contributions to the mitigation of CH_4_ and CO_2_ emissions.

CH_4_ has a global warming potential 28 times higher than that of CO_2_ over a 100-year period^46^. As a result, the RS + CF treatment had the highest net GWP, mainly due to the greater GWP from CH_4_ than from CO_2_. In contrast, the lowest net GWP was found in the BC + CF treatment because of the low GWP value of CH_4_ (Table 1). This indicated that the GWP of CH_4_ played a more significant role in reducing the net GWP than the GWP of CO_2_ in this study. Naser et al*.*^47^ also illustrated that the GWP of CH_4_ dominated the net GWP of rice paddies. The GWP of CH_4_ accounted for 71.9–86.1% of the annual net GWP, while the GWPs of CO_2_ and nitrous oxide (N_2_O) accounted for 13.8–26.5% and 0.13–1.61%, respectively. When the GHG emissions were yield-scaled, the GHGI was significantly lower in the BC + CF treatment than in the RS + CF treatment (*p* < 0.01; Table 1); moreover, there was no significant difference in rice grain yield (*p* < 0.01; Table 2) because the BC + CF amendment decreased the net GWP by 46.8% compared with that in the RS + CF treatment. Furthermore, the GHGI in the RS + CF treatment was significantly lower than that in the CF treatment (*p* < 0.01; Table 1) because the increase in rice grain yield caused by the RS + CF treatment can offset its relative ability to mitigate GHG emissions.

The present study demonstrated that the RS + CF and BC + CF amendments significantly increased in rice grain yield (*p* < 0.01; Table 2). Comparing these amendments to the application of CF alone, the grain yield increased by 68.3% and 87.3%, respectively. Similar results were reported in previous studies, indicating that increased phosphorus (P) and potassium (K) availability in soils treated with rice straw-derived biochar^25^ and improved soil total C after rice straw and its derived biochar amendment^21^ resulted in enhanced rice grain yield. The SOC plays a central role in enhancing nutrient availability in the soil, significantly influence on the delivery of nutrients to plant roots, and consequently serving as a pivotal factor in promoting crop yield^48^. The increased soil TOC (Fig. 1b) and the P and K contents in the RS and BC materials (Table 4) reflected these results.

In conclusion, the incorporation of leftover rice straw (10 t ha^−1^) increased the seasonal cumulative CH_4_ emissions and net GWP. This increase was attributed to elevated contents of DOC and MBC in the soil, which subsequently led to higher abundances of archaea and bacteria. In contrast, the application of biochar (3 t ha^−1^) obtained from the pyrolysis of leftover rice straw has significant potential for reducing seasonal cumulative CH_4_ emissions, net GWP, and GHGI. The reduction in CH_4_ emissions attributed to the amendment of rice straw-derived biochar did not elevate soil levels of DOC and MBC, causing a reduction in archaeal abundance. Additionally, it led to an increase in soil TOC content. Furthermore, rice straw and biochar amendments enhanced rice grain yield. Our findings indicated that using biochar derived from leftover rice straw is an advantageous strategy for mitigating CH_4_ emissions, net global warming potential, and greenhouse gas intensity. Furthermore, this approach has potential for enhancing crop productivity in tropical rice cultivation.

## Methods

### Site characteristics and experimental design

The experimental field was established in Ban Non Muang, Tambon Sila, Mueang Khon Kaen, Khon Kaen, Thailand (48Q 265,026 1,825,361). The paddy field soil was classified as Aquic Haplustalfs according to the Key to Soil Taxonomy^49^. The initial topsoil (0–20 cm) was classified as loam texture with sand (50.3%), silt (33.8%), and clay (15.9%) with SOM content of 0.7%, a SOC content of 0.4%, total nitrogen (N) content of 0.04%, pH (1:5) = 6.3, and bulk density of 1.5 g cm^−3^. The average temperature conditions during the field trial period varied between 24 and 33 °C, and the average rainfall was 4.25 mm day^−1^. The field experiment was conducted during the rice cultivation season (June–November) of 2021. The experimental design was a randomized complete block design with three replicates on a plot measuring 5 m × 5 m. Three treatments were adopted in this experiment: (1) chemical fertilizer (CF), (2) chemical fertilizer with rice straw applied at 10 t ha^−1^ dry weight (RS + CF), and (3) chemical fertilizer with rice straw-derived biochar applied at 3 t ha^−1^ dry weight (BC + CF). The residual rice straw rate was equal to 10 t ha^−1^ based on the average amount of rice straw remaining after harvest in the field^32,33^. This study employed rice straw-derived biochar at 3 t ha^−1^, which was derived from the 10 t ha^−1^ of rice straw that remained after the pyrolysis of rice straw. The residues were incorporated into the soil at a depth of 0–15 cm and incubated for 4 weeks before rice transplantation. The application rate of chemical fertilizers was determined based on the rice requirements (RD6 variety). The total amounts of N, P, and K used in this study were 188, 38, and 132 kg ha^−1^, respectively. All P and K with half of the N were applied at 7 days after transplanting (DAT), and the remaining half of the N was applied at the early panicle initiation stage (70 DAT). Urea, diammonium phosphate, and muriate of potash were used as sources of N, P, and K, respectively. The soils in all the plots were submerged for 10 days before transplanting. Rice seedlings (28 days old) of the RD6 variety were transplanted in July at a hill density of 20 cm by 20 cm. During the experimental period, the plot water level was maintained at 5–7 cm above the soil surface until 1 week before the harvest period. Weeds were controlled by hand hoeing at the tillering stage, and pesticides were used in the field to control rice pests and diseases.

### Rice straw and rice straw-derived biochar properties

Rice straw (RS) was collected from the cultivated experimental field, air-dried and cut into pieces 5–10 cm in length. Biochar (BC) was produced from rice straw via pyrolysis at 350 °C under oxygen-limited conditions in a 200 L traditional drum kiln, which is commonly used in northeastern Thailand^34^. The chemical characteristics of the RS and BC are shown in Table 4.

### Soil sampling and analysis

Soil samples were collected at a depth of 0–20 cm from the soil surface in each plot on the first day after transplanting (1 DAT) or at the seedling (SL) stage, tillering (TL) stage at 56 DAT, panicle initiation (PI) stage at 105 DAT, and harvest (HV) stage at 119 DAT for chemical and biological property analysis. The soil samples were sealed in plastic zip-lock bags, transported to the laboratory, and stored in a refrigerator at − 4 °C prior to biological analysis; the samples for chemical analysis were subsequently air-dried. The soil pH and redox potential (Eh) were determined in the field using a portable pH/mV/°C meter (HANNA Instruments, HI8424). The TOC content was determined in air-dried soil samples by dry combustion using a TOC/TN analyzer (Multi N/C 2100s, Analytik Jena, Jena, Germany). The soil dissolved organic carbon (DOC) and microbial biomass carbon (MBC) contents were determined in field-moist soil samples using the chloroform fumigation-extraction method^50^. For the nonfumigated sample, 10 g of soil was extracted with 40 ml of 0.025 M K_2_SO_4_ on a horizontal shaker at 250 rpm for 30 min, and the samples were subsequently centrifuged at 4560 rpm for 30 min. For the fumigated sample, 10 g of soil was fumigated with ethanol-free chloroform for 24 h at room temperature in a desiccator and subsequently extracted in the same way^51^. The supernatants were analyzed for TOC using a TOC/TN analyzer. The MBC was calculated as the difference in the values of fumigated and non-fumigated samples using a *k*_EC_ factor of 0.45^52^. The DOC content was estimated using the TOC contents of the nonfumigated samples.

### CH_4_ and CO_2_ analysis

Gas samples were collected via a closed chamber method, and the chamber had a size of 60 cm width × 60 cm length × 80 cm height. The chamber was placed in a 1 m × 1 m area in the middle of each plot. The daily gas samples were collected once a week in the morning by using a 10 ml syringe at 0, 10, 20, and 30 min after the chambers were placed on the fixed frames, and the samples were stored in 5 ml evacuated vials. The air temperature inside the chamber and the water level were monitored during gas collection. Gas samples were analyzed for CH_4_ and CO_2_ concentrations by an Agilent 7890B (Agilent Technologies, Inc., USA) gas chromatograph equipped with a flame ionization detector (FID) operated at 300 °C, and a HaySep Q packed column was used. N_2_ was used as the carrier gas for the FID at a flow rate of 20 ml min^−1^. The cumulative CH_4_ and CO_2_ emissions were calculated from the daily emission rates over the rice growing season.

### Global warming potential and greenhouse gas intensity

The net global warming potential (net GWP) (kg CO_2_-eq ha^−1^ season^−1^) was computed using a 100-year time horizon as suggested by the IPCC^46^ from the GHG emissions of each field (conversion factors of 1 for CO_2_ and 28 for CH_4_). The estimations of the GWP of CH_4_, the GWP of CO_2_, and the net GWP are as follows:

1 $${\text{GWP}}\;\;{\text{of}}\;\;{\text{CH}}_{{4}} = {\text{CH}}_{{4}} \times {28}$$

2 $${\text{GWP}}\;\;{\text{of}}\;\;{\text{CO}}_{{2}} = {\text{CO}}_{{2}} \times {1}$$

3 $${\text{net}}\;\;{\text{GWP}} = {\text{CH}}_{{4}} \times {28} + {\text{CO}}_{{2}} \times {1}$$

where CH_4_ represents the seasonal CH_4_ emissions (kg ha^−1^) and CO_2_ represents the seasonal CO_2_ emissions (kg ha^−1^).

The greenhouse gas intensity (GHGI) (kg CO_2_-eq t^−1^ GY) was determined by the following equation^53^:

4 $${\text{GHGI}} = {\text{net}}\;\;{\text{GWP}}/{\text{rice}}\;\;{\text{grain}}\;\;{\text{yield }}\left( {{\text{t}}\;{\text{ha}}^{{ - {1}}} } \right)$$

### DNA extraction and microbial abundance analysis

Total soil DNA was extracted from 0.25 g fresh soil using DNeasy PowerSoil Pro Kits (Qiagen, Hilden, Germany), and the samples were examined using quantitative polymerase chain reaction (q-PCR) to determine archaeal and bacterial abundances. Using SLAN-96P real-time PCR instrument (SANSURE BIOTECH INC, China), q-PCR of the bacterial 16S rRNA gene and the archaeal 16S rRNA gene was carried out using primer combinations that target the 16S rRNA genes of bacteria and archaea. q-PCR of the bacterial 16S rRNA gene and archaeal 16S rRNA gene was performed using a SLAN96P real-time PCR system. The primer pairs targeting the bacterial 16S rRNA gene and archaeal 16S rRNA gene consisted of the forward primer Eub338 (5′-ACTCCTACGGGAGGCAGCAG)^54^ and reverse primer Eub518 (5′-ATTACCGCGGCTGCTGG)^55^ and the forward primer Ar109 (5′-ACKGCTCAGTAACACGT) and reverse primer Ar912 (5′-CTCCCCCGCCAATTCCTTTA)^56^; these were used for the bacterial 16S rRNA gene and archaeal 16S rRNA gene, respectively. The q-PCR methodology was based on Kumputa et al*.*^34^. A total of 25 µL of PCR mixtures was used, each containing 12.5 µL of EXPRESS SYBR® GreenERTM (Invitrogen, Carlsbad, CA, USA), 0.4 µM primer, 1 µL of 10 ng µL^−1^ DNA template, and ultrapure water to a volume of 25 µL. All the samples were examined in duplicate. The reaction conditions included a 10-min initial denaturing phase at 95 °C, 40 cycles of 30 s of denaturing at 95 °C and 30 s of primer annealing at 55 °C and 57.5 °C for the primers Eub338/Eub518 and Ar109/Ar912, respectively, and final 45 s of primer extension at 72 °C. The DNA copy numbers of the 16S rRNA genes per g of dry soil used to measure the abundances of bacteria and archaea using q-PCR were elaborated.

### Measurements of rice growth and yield

Rice yield and biomass were estimated by harvesting rice from an area of 1 m × 1 m in each plot. The parameters considered were the number of tillers, plant height, straw yield, filled grain percentage, 1000-grain weight, and grain yield. Tillers were physically counted from 25 marked hills in an area of 1 m × 1 m in each plot at the maximum tillering stage (56 DAT). The plant height was measured from the ground surface to the highest point of the plant using a tape measure at the harvest stage (119 DAT). Five samples of 1000 grains in each plot were taken randomly from the filled grains and weighed to record the 1000-grain weight. The percentage of filled grains was calculated by dividing the number of filled grains by the total spikelet number. For laboratory analyses, the rice straw and grains collected from each pot were oven-dried at 60 °C until the weight of the plants was constant.

### Statistical analysis

The data were analyzed for statistically significant differences using ANOVA and least significant difference (LSD) tests at the 0.01 probability level. Pearson’s correlation was used to determine the relationships between the cumulative GHG emissions and soil properties at the tillering stage. We used Statistix 10 software and IBM SPSS Statistics software (version 28) to carry out the statistical tests.

### Ethical statement

This study does not include human or animal subjects. The plant collection and use was in accordance with all the relevant guidelines.

## Data Availability

The datasets generated during and/or analyzed during the current study are available from the corresponding author on reasonable request (P.L., phrula@kku.ac.th).
